# Tumeur sous scapulaire: élastofibrome dorsal bilatéral à propos d'un cas

**DOI:** 10.11604/pamj.2015.21.43.6865

**Published:** 2015-05-21

**Authors:** Meriem Dlimi, Samira Boukind, Oumkeltoum Elatiqi, Driss Elamrani, Yassine Benchamkha, Saloua Ettalbi

**Affiliations:** 1 Service de Chirurgie Plastique, Reconstructrice, Esthetique et Brules, CHU Mohamed VI, Marrakech, Maroc

**Keywords:** Tumeur, scapula, parties molles, benigne, bilatéral, élastofibrome, dorsal, Tumor, scapula, soft tissue, benigne, bilateral, elastofibroma, dorsal

## Abstract

L’élastofibrome dorsal est une tumeur bénigne rare des parties molles siégeant typiquement sous la pointe de la scapula. Nous rapportons notre observation; pour préciser les caractéristiques cliniques et paracliniques de ce type de tumeur ainsi que les modalités de prise en charge; cette entité gagnerait à être connue par tout praticien; pour permettre une attitude thérapeutique bien codifiée.

## Introduction

L’élastofibrome a été décrit pour la première fois par Jarvi et Saxen en 1959 lors du 12ème congrès d'anatomopathologie scandinave et rapporté en 1961 [1, 2]. L’élastofibrome est une tumeur bénigne, rare et peu connue [3, 4], qui se présente comme une tumeur non encapsulée [3]. C'est une tumeur localisée typiquement au niveau de la paroi thoracique postérieure et précisément au niveau des parties molles de la pointe de la scapula bordée par le muscle grand dorsal et le muscle serratus antérieur [3, 5–8]. Nous rapportons l'observation d'une patiente qui présentait un élastofibrome dorsal bilatéral afin de préciser les caractéristiques cliniques et paracliniques de cette tumeur; ainsi que les modalités de prise en charge thérapeutique.

## Patient et observation

**Figure 1:**
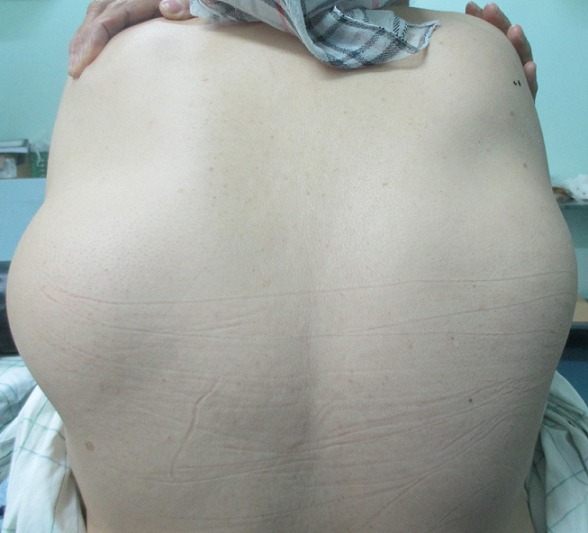
Vue de face montrant les deux masses scapulaires

**Figure 2:**
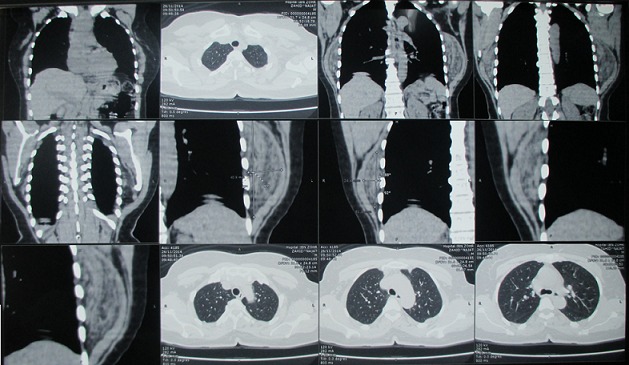
Les mensurations des deux masses sous scapulaires sur la TDM

**Figure 3:**
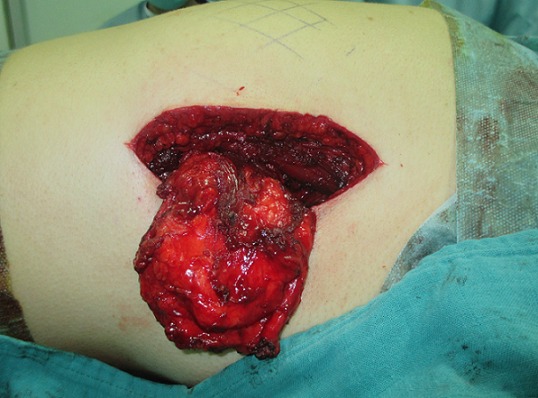
Exérèse des deux masses sous scapulaires

**Figure 4:**
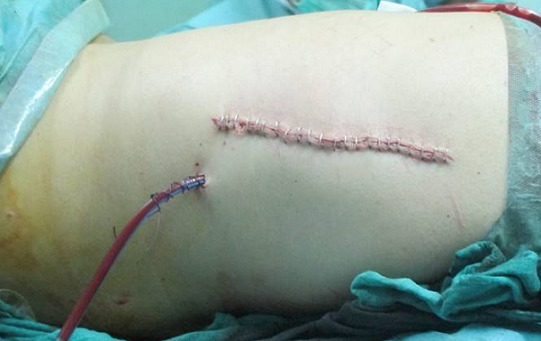
Aspect en post opératoire immediate

**Figure 5:**
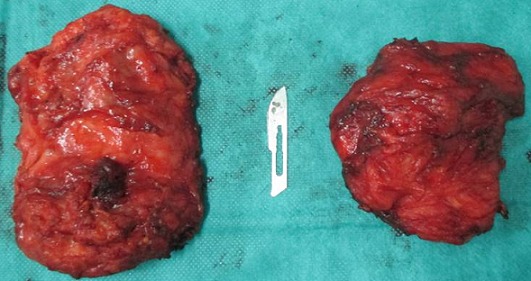
Les deux masses après exérèse

Mme N.Z, âgée de 55 ans sans antécédents pathologiques particuliers, s'est présentée pour une tuméfaction de la région scapulaire droite et gauche évoluant depuis quatre ans, augmentant progressivement de volume et devenant de plus en plus gênante lors de la mobilisation de l’épaule. L'examen clinique mettait en évidence une tuméfaction bien limitée de 12 cm de grand axe en regard de la pointe de la scapula gauche et se prolongeant en sous-scapulaire; cette masse était mieux visible en abduction et en antépulsion du membre supérieur gauche, de consistance ferme, douloureuse à la palpation, mobile par rapport au plan superficiel et profond de la pointe de la scapula. A droite, mise en évidence d'une masse de 9 cm de grand axe,bien limitée, siégeant au niveau de la pointe de la scapula droite, de consistance ferme, indolore à la palpation, mobile par rapport aux deux plans superficiel et profond, et elle devient plus visible si on met le membre supérieur droit en abduction et en antépulsion. Pas d'adénopathies axillaires palpables ni à droite ni à gauche. La mobilité des deux épaules était normale en dehors d'un pseudo-claquement lors de l'abaissement et de la rétropulsion du membre supérieur (Figure 1). L'examen n'objectivait pas de troubles vasculonerveux au niveau des deux membres supérieurs. L’état général de la patiente était conservé; le bilan biologique était normal. La radiographie standard et l’échographie des deux masses n'ont pas pu être faites. La TDM thoracique (Figure 2) a été demandée afin de déterminer la constitution de la tumeur, ses mensurations, son extension et ses rapports avec la scapula et la cage thoracique. L'examen montrait la présence d'une masse pariétale de la région scapulaire gauche située entre le muscle grand dorsal et le grill costal mesurant 112mm, 82mm, 40 mm, à double composante fibreuse et graisseuse, sans calcifications. Cette masse présente des contours réguliers sans infiltration du muscle grand dorsal ou endothoracique en regard. Il existe également une deuxième masse controlatérale symétrique de même situation et de mêmes caractères tomodensitométriques, de taille moins importante mesurant 80mm, 60mm, 26mm de diamètres, absence de lésion parenchymateuse pulmonaire nettement visible, absence d'adénopathies médiastinales, absence d'anomalie des gros vaisseaux du médiastin, absence d’épanchement pleuropéricardique et absence de lésion osseuse thoracique. L'IRM n'a pas pu être faite. La patiente a bénéficié d'une exérèse chirurgicale des deux masses sous scapulaires droite et gauche (Figure 3). L'intervention s'est déroulée sous anesthésie générale en décubitus ventral. La voie d'abord était oblique au bord externe de la masse; parallèle aux fibres musculaires du muscle grand dorsal puis perpendiculairement aux fibres du muscle serratus antérieur, la tumeur a été emporté en masse, adhérente à la face profonde du muscle grand dorsal. Une hémostase soigneuse a été assurée en fin d'intervention ainsi qu'un drainage aspiratif au niveau des deux côtés droit et gauche (Figure 4). La masse à gauche pesait 200g et à droite 130g (Figure 5). Une immobilisation par écharpe à titre antalgique a été préconisée pendant une semaine. Les suites opératoires étaient simples avec récupération de la mobilité de l’épaule et disparition de la gêne fonctionnelle. L’étude anatomopathologique a confirmé le diagnostic d’élastofibrome et l'exérèse totale de la tumeur.

## Discussion

L’élastofibrome dorsal bilatéral est une tumeur bénigne, rare et d’évolution lente. Elle survient chez 2% des personnes âgées de plus de 60 ans [9]. Elle siège le plus souvent au niveau de la région infra et périscapulaire et presque exclusivement adjacente à la pointe de la scapula [6, 10, 11]. Cependant, d'autres localisations ont été rapportées [6]: olécranienne, ischiatiques, interdigito-plantaires, digitales, deltoïdienne, axillaire, trochantérienne, etc. C'est une tumeur qui se rencontre avec prédilection chez les sujets de plus 55 ans, plus souvent de sexe féminin [5, 12]. Ainsi, la série de Nagamine et al, de 170 patients retrouve 158 femmes pour 12 hommes [8]. Néanmoins, un cas d’élastofibrome a été rapporté chez l'enfant [13]. Cliniquement, la masse est asymptomatique dans la moitié des cas; 25% des patients rapportent un sentiment de gêne ou de raideur lors de la mobilisation de l’épaule [6, 9, 11, 14]. Une symptomatologie douloureuse scapulaire ou sous scapulaire n'est observée, que dans 10% des cas. L'atteinte neurologique du membre supérieur peut être exceptionnellement observée évoquant une névralgie cervico-brachiale. La localisation bilatérale de l’élastofibrome dorsal est peu fréquente avec un développement asynchrone des deux masses. La possibilité de deux localisations différentes chez le même patient [10, 11]. L'examen clinique met en évidence une masse ferme, fixée aux plans profonds, mobile par rapport aux plans superficiels et sans signes d'infiltration cutanée. Elle est le plus souvent indolore, palpable, bien visible pour les localisations sous scapulaires, surtout lorsque le membre supérieur est en antépulsion avec abduction. Le bilan biologique est presque toujours normal. La radiographie du thorax peut montrer une surélévation de la scapula et un élargissement de l'espace scapulothoracique. Une opacité interscapulothoracique peut-être mise en évidence, mais sans lyse osseuse ou calcification associée [6]. La tomodensitométrie thoracique met en évidence une masse de densité identique que celle des tissus mous avoisinants, avec des zones de moindre densité [15]. La tumeur est souvent mal-limitée et non homogène. L'IRM objective une masse souvent hétérogène, bien définie, révélant deux signaux différents en pondération T1 l'un d'une intensité intermédiaire équivalent à celui des muscles squelettiques, le second de haute intensité correspondant à la graisse emprisonnée au sein de la masse. En T2, on observe une augmentation de l'intensité du signal. L'injection du gadolinium ne rehausse pas le signal [15]. Ces examens paracliniques ont pour objectifs de déterminer les mensurations de la masse, son siège exact par rapport aux structures musculaires adjacentes. Seule la biopsie de la masse éventuellement effectuée à l'aiguille permet le diagnostic de certitude en montrant des fibres élastiques de morphologie caractéristique au sein de tissus fibreux et adipeux. Le traitement des formes symptomatiques et douloureuses de l’élastofibrome dorsal est l'exérèse chirurgicale complète. Pour certains auteurs, même en absence de manifestation clinique lorsque le diamètre est supérieur à 5 cm, il faut réaliser une résection chirurgicale. Pour d'autres, du fait de l'absence de transformation maligne, seule la biopsie de confirmation du diagnostic s'impose en l'absence de symptomatologie [6, 9, 11, 14].

## Conclusion

L’élastofibrome dorsal bilatéral est certes une tumeur bénigne rare et d’évolution très lente, mais à connaitre pour l’évoquer devant une masse sous scapulaire gênant la mobilisation de l’épaule chez une femme de plus de 55 ans et nécessitant après les examens paracliniques notamment une tomodensitométrie thoracique, si elle est symptomatique, une exérèse chirurgicale, avec pratiquement pas de risque de récidive.
